# Populismus und Bildung

**DOI:** 10.1007/s40955-020-00173-0

**Published:** 2020-11-19

**Authors:** Ekkehard Nuissl, Katarina Popović

**Affiliations:** 1 Universität Kaiserslautern, Kaiserslautern, Deutschland; 2 Universität Belgrad, Belgrad, Serbien

Populismus ist als ein Phänomen der politischen und gesellschaftlichen Gegenwart allgegenwärtig, wirkmächtig und polarisierend. Für manche ist „Populismus“ ein Kampfbegriff in der politischen Arena, für manche die begriffliche Fassung krisenhafter demokratischer Strukturen. Wir stellen Populismus in den unterschiedlichsten staatlichen, politischen und sozialen Kontexten fest – und zwar überall in der Welt. Regierende Personen, wie der amerikanische Präsident Trump, der brasilianische Präsident Bolsonaro, der türkische Präsident Erdogan gelten als Populisten, auch europäische Politiker wie der englische Premierminister Johnson, der ungarische Premier Orbán und der serbische Präsident Vučić. Die von ihnen vertretene und durch sie realisierte Politik ist populistisch. Sie behaupten, das Interesse des Volkes (lat. *populus*) zu repräsentieren: „Make America great again.“ Oder ein freies United Kingdom könne zu alter Größe finden. Und so fort. Die Video-Serie „America First, every other country second“ etwa ist eine globale, urkomische Antwort auf die populistischen Botschaften des Präsidenten Trump.^1^

Es ist nicht so, dass sich Populismus vor allem in autoritären Regierungsformen zeigt, etwa in ehemaligen sozialistischen Ländern, asiatischen Ländern mit autoritären Traditionen, Ländern mit kolonialer Leidensgeschichte. Es zeigt sich, dass auch Länder mit langer demokratischer Tradition anfällig für Populismus sind, denn sogar in gefestigten europäischen Demokratien verbuchte Populismus, vor allem Rechts-Populismus, beachtliche Erfolge.

Sichtbar ist Populismus vor allem in politischen Aktionen, die im öffentlichen Raum organisiert sind – Protestkundgebungen, Aufmärsche, Ansammlung verschiedener Gruppen. Häufig richten sind solche Aktionen in den letzten Jahren gegen Migrantinnen und Migranten, die „als Angriff auf die ‚heile Welt‘ ethnokultureller Beschaulichkeit interpretiert“ werden, wie eine Studie der TU Dresden zeigt (MIDEM 2018, S. 80). Populismus ist sichtbar geworden, mit unterschiedlichen Zielen. Populismus zeigt sich selbstsicher im öffentlichen Raum. Parteien suchen nach Popularität, und nicht nur „rechte“ Parteien greifen nach populistischen Mitteln, um sie zu gewinnen. Insbesondere „rechte“ Parteien aber haben in Deutschland und andernorts (etwa Frankreich, Niederlande, Österreich) mittels populistischer Taktiken für eine diskursive Verschiebung der öffentlichen Debatten zu den wichtigsten politischen und wirtschaftlichen Entscheidungen beigetragen.

Populismus zeigt ein enormes Potenzial zur Variation von Kontext, Zielgruppen, Inhalt und Themen. Er praktiziert in unterschiedlichen Bereichen sein Instrumentarium und sein Narrative. Beispiele zeigt die COVID-19-Krise; Politiker der USA, Brasilien, Bolivien und Serbien haben sie genutzt, um sich dem „Volk“ als mächtig und unerschrocken zu zeigen, und verteufeln „Schuldige“ und „Feinde“ – die WHO, die Wissenschaft, die Chinesen.

Das Phänomen des Populismus ist ein Symptom, mehr noch: ein wichtiger Indikator für grundlegende Probleme der repräsentativen Demokratie. Es tritt in unterschiedlichen, kontextabhängigen Erscheinungen auf, hat unterschiedliche Ursachen und Wirkungen. Dies macht die Definition dessen, was „Populismus“ ist und was unter dem Attribut „populistisch“ verstanden werden kann, außerordentlich schwierig.

In der Wissenschaft werden verschiedene Spielarten von Populismus beschrieben: „Anti-Establishment“, „Pro-Volkssouveränität“ und „Anti-Pluralismus“ (z. B. Akkerman et al. 2013; Hawkins et al. 2012). Diese werden empirisch getestet (z. B. Vehrkamp und Merkel 2018). Wielenga und Hartleb (2011) unterscheiden vier Dimensionen, hier umgestellt und inhaltlich modifiziert:

- die *technische* Dimension, indem der Populismus vereinfacht und einen Gegensatz zwischem dem als homogen konstruierten Volk und eines „Äußeren“ (Elite in den Machtpositionen, Minderheiten, Migranten etc.) behauptet;
- die *emotionale* Dimension, indem der Populismus Gefühle und Ängste anspricht, meist über eine charismatische Figur, die im Namen des Volkes gegen (das ist hier einzusetzen) kämpft;
- die *mediale* Dimension, in der Schlagzeilen, Events und Personen hochgespielt werden (negative wie positive), Aufmerksamkeiten erzeugt und bedient werden;
- die *inhaltliche* Dimension, indem der Populismus Gegenposition zu unterschiedlichsten Sachverhalten und Ideologien bezieht, etwa den „Globalkapitalisten“ (die auch bei den aktuellen Verschwörungstheorien der Corona-Krise eine Rolle spielen) oder den Migrantinnen und Migranten oder dem Islam.

Der Begriff „Populismus“ wird zwar unterschiedlich akzentuiert, die Dimensionen werden jeweils unterschiedlich gewichtet (Ionescu und Gellner 1969), relativ einvernehmlich aber wird die erste, die *technische *Dimension als Wesensmerkmal des Populismus begriffen, die Konstruktion des guten „Wir“ gegenüber einem schlechten „die Anderen“. Das gute „Wir“ ist dabei immer das Volk. Es wird zu einem homogenen Ganzen verklärt, in dem die „einfachen Leute“ und der „gesunde Menschenverstand“ das Subjekt und den obersten Wert darstellen. „Die Anderen“ sind austauschbar, je nach Kontext, Aktualität und Problem. Meist sind die Anderen – im politischen Kontext – „die da oben“, eine ebenfalls konstruierte (Macht‑)Elite. In dieser Variante ist der Populismus Anstoß für eine grundlegende Demokratiedebatte (Bohmann et al. 2018), darauf kommen wir zurück.

Die „Anderen“ können aber auch Minderheiten sein. In einigen Ländern Europas sind diese seit einigen Jahren die Geflüchteten und Migrantinnen und Migranten, aber auch die Homosexuellen in Polen, die Sinti und Roma in Rumänien und anderen Ländern Südosteuropas, die Muslime und wieder die Juden. In dieser Variante ist Populismus eher Produzent oder Unterstützer eines gesellschaftlichen Schismas. Diese beiden Varianten sind die Hauptlinien dessen, was man unter „Rechtspopulismus“ versteht (Hartleb 2004).

Der „Linkspopulismus“ bedient das gleiche Modell: hier das „Wir“ der „Guten“, da die „Anderen“, also die „Bösen“. Dabei wird jedoch ein Klassenmodell zugrunde gelegt, die „gute“ Arbeiterklasse und die „bösen“ Kapitalisten und ihre Helfershelfer (Mouffe 2018). Linkpopulismus teilt mit den Rechtspopulisten „anti-elitist sentiments“, setzt sich aber für Pazifismus und soziale Gerechtigkeit ein und richtet sich gegen Globalisierung. Autoren wie Mouffe versuchen, Linkspopulismus zu rehabilitieren und dessen Strategien in den Rahmen der pluralistischen Demokratie zu integrieren, wobei die Prinzipien des politischen Liberalismus bewahrt blieben, und behaupten,

that democratic discourse plays a crucial role in the political imaginary of our societies. And through the construction of a collective will, mobilizing common affects in defence of equality and social justice, it will be possible to combat the xenophobic policies promoted by right-wing populism (Mouffe 2018).

Diese Charakterisierung von Populismus bedient wesentliche Bedürfnisse der Menschen. Historisch entstand er auch auf der Grundlage dieser Bedürfnisse (Canovan 1981). An erster Stelle steht dabei das Bedürfnis nach Zugehörigkeit und nach Identität. Die Konstruktion des „Wir“, so brüchig sie auch ist, gibt vielen Menschen eine Art soziales Zuhause. Es ist gekennzeichnet durch gemeinsame Werte, durch tradierte und nicht hinterfragte Werte, deren Akzeptanz Sicherheit schafft. Verbunden ist dies mit der Frage der Identität des Individuums innerhalb einer sozialen Gemeinschaft, eine *sine qua non* jeder individuellen Identität auch im Falle einer eigenen Absonderung. Dieses Bedürfnis akzeptierte etwa die in Deutschland mehrfach unternommene Suche nach einer deutschen „Leitkultur“, die eine Abgrenzung des „Wir“ ebenso wie die erhoffte Pflicht einer Integration in dieselbe ermöglicht.

Die Rückbesinnung auf eine Wertegemeinschaft des „Volkes“ ist in Zeiten des Wandels und der Globalisierung von wachsender Bedeutung (Lucardie 2011). Der gesellschaftliche Wandel findet in immer rascheren Schüben statt und führt zu „Modernisierungskrisen“, die in Ungleichzeitigkeiten der Akzeptanz und Anpassung liegen. Die Globalisierung hat unter anderem zu einer wachsenden Zahl von Kosmopoliten, also „Weltbürgern“, geführt, die sich sprachlich und kulturell in unterschiedlichen sozialen Umwelten bewegen können. Diese werden von den „Heimbürgern“ als ähnlich fremd empfunden wie etwa Migranten und als ähnlich elitär wie die politischen Eliten.

In der Wertegemeinschaft ist diese im Wandel begriffene Spannung zwischen Außen und Innen, Nah und Fern, Oben und Unten immer eine Gefahr für die Sicherheit und Identität der Menschen, die weniger sachlich als vielmehr sozialpsychologisch zu erklären ist. Denn Emotionen, dieser Standpunkt hat in den letzten Jahren in der politischen Wissenschaft immer mehr an Bedeutung gewonnen, sind in Bezug auf Werte wie Heimat und Sicherheit, wirkmächtiger als Fakten und Sachverhalte (Arnold 2016).

Mit den *Emotionen* ist die zweite der genannten Dimensionen angesprochen. Populismus spricht gezielt Emotionen an, Ängste, Gefühle, Abneigungen, Unsicherheiten und Komplexe, aber auch Stolz, Ehrgeiz und Kameradschaft (Giroux 2020; Müller 2016). Giroux nimmt an, dass die Ungleichheiten und Ungerechtigkeiten, welche die Wirtschaft in die Weltordnung bringt, hier die Hauptprobleme sind:

[A] rise of a kind of right-wing populism that merges the elements of White supremacy with the fear and anxiety that comes out of a new global order that is based on productivity and the constant mobilization of wealth offered to the one percent (Giroux 2020).

Die emotionale Lücke, welche in rational aufgebauten Repräsentationsstrukturen besteht, wird von den Populisten genutzt und bedient. Das Wir-versus-die-Anderen-Schema ist im Kern kein sachliches Konzept, sondern eines, das soziale Sachverhalte in sozialpsychologische Phänomene transformiert. Die Ansprache der Emotionen erfolgt auf unterschiedliche Weise, etwa in einer Polarisierung, die zu Kommunikationsabbruch und Kampfesstimmung führt. Oder in symbolischen Interaktionen, in Zuschreibungen, die möglicherweise sachlich falsch, emotional aber wirksam sind. Die Strategie des Populismus, Sachverhalte zu vereinfachen, auch verzerrend zu vereinfachen, ist dabei ein wichtiger Bestandteil, den durchaus auch als unpopulistisch geltende Politiker nutzen – etwa Minister Rüttgers mit seiner Antimigrationsformel „Kinder statt Inder“ (Kerner 2008, S. 343 f.).

Zur Emotionalität gesellt sich auch die Tendenz des Populismus, zu moralisieren. Meist werden die Moralbegriffe aus dem geschöpft, was als Wertekanon des (künstlich homogenisierten) Volkes gelten kann. Solche moralisierenden Appelle werden nicht als abstrakte Regeln oder Richtlinien formuliert, sondern direkt ad populum, ad hominem adressiert. Und personalisiert. Und das mit Folgen. Ein Beispiel ist die populistische Brandmarkung von Migranten, Flüchtlingen und Ausländern als Kriminellen, Vergewaltigern und Dieben. Bei der Aufklärung der Fälle wird nun in der Regel – in Folge dieses populistischen Bias – die Herkunft der möglichen Täter thematisiert. Oder ein anderes Beispiel: Bill Gates, als Multimilliardär ohnehin Teil der herrschenden Machtelite, steht unter dem Verdacht, verschwörungstheoretisch, die Weltregierung an sich reißen zu wollen – mit der Folge, dass dieser Verdacht ständig zu entkräften ist.

Solche Moralisierungen sind ein Instrument, die Gunst des „Volkes“ zu suchen und zu finden. Das ist bereits eine belastbare Erkenntnis aus der Frühzeit der Massenpsychologie (vgl. G. Le Bon 2016). Allgemeine Wertvorstellungen werden vereinfacht und moralisch aufgeladen, sie sind ohne intellektuelle Barrieren vermittelbar.

An die Stelle programmatischer Positionen setzen populistische Aktivitäten oft Appelle an die Werte des Volkes, der einfachen Leute, wie „gesunder Menschenverstand“, Stärke, Fleiß. „Demnach werden anstelle eines weltanschaulichen Gesamtkonzepts kontextabhängige politische Forderungen um ein schwach ausgebildetes moralisches Kernkonzept gruppiert“ (Lewandowsky 2011, S. 220). Diese schwarz-weiße Sichtweise ermöglicht den Menschen, die Komplexität der realen Welt zu bewältigen und gibt ihnen das beruhigende Gefühl, das Gute erkannt zu haben, auf der guten Seite zu stehen und die richtigen Werte zu verteidigen.

Schließlich findet sich auf dieser Dimension auch die tendenzielle Wissenschaftsferne oder gar -feindlichkeit des Populismus. Immer vor dem Hintergrund der Polarisierung von „Volk“ und „Elite“ rücken die sachlich und differenziert argumentierenden Wissenschaftlerinnen und Wissenschaftler auf die Seite der Elite, da sie „abgehoben“ und gegen den „gesunden Menschenverstand“ zu agieren scheinen, ganz abgesehen vom Verdacht der Korrumpierbarkeit durch die Machtelite. Gegenbeispiele wie aktuell der Charité-Virologe Christian Drosten, der regelmäßig im Fernsehen Kommentare zur Lage abgibt und breit akzeptiert ist, sind seltene Ausnahmen, wobei sicher eine Rolle spielt, dass sie sich einfacherer Worte und Bilder bedienen und populistische Wege (wie hier das TV) nutzen.

Die dritte Dimension des Populismus ist die *mediale *Präsenz. Dörner spricht hier von einem „Politainment“, einer Mischung von Politik und Unterhaltung. Sie realisiert sich als mediengerechte Theatralisierung, als „Event-Politik“, als „Image-Politik“ (Dörner 2001).

In der Tat spielt die Möglichkeit, die heutigen Medien, das sind die Massenmedien ebenso wie die sozialen Medien, in ihrer Vielzahl und Vielfalt zu nutzen, eine große Rolle beim Erstarken populistischen Stils. Sie ist aber keine *conditio sine qua non* des Populismus – schon die erste „populist party“ in den USA (1891–1908) wandte entsprechende Stilmittel an. Die heutigen Medien ermöglichen jedoch in weit größerem Maße, nicht nur breite Gruppen anzusprechen und zu agitieren, sondern auch über spezifische Kanäle Gleichgesinnte zu finden, Erfahrungen auszutauschen und sich gegenseitig zu motivieren. Solche „ideologischen“ Kanäle existieren für alle Arten von (auch inhumanen) Werten, Ideen und Richtungen.

Kaum ein Ereignis hat in der neueren Zeit so viele Leute weltweit mobilisiert wie Covid-19 – die Zahl der falschen Nachrichten („fake news“) und Verschwörungstheorien ist kaum übersehbar (Hendricks 2019). Und ihre Verfechter nutzen die Medien auf intensive Weise. „Verschwörungstheoretiker“ der Corona-Krise etwa tummeln sich auf dem Messanger-Dienst Telegram^2^. Populismus, so Mudde (2004), folgt dem Zeitgeist und nutzt ihn, bedient sich der Funktionslogik der Mediendemokratie.

Die vierte, die *inhaltliche* Dimension schließlich wird sehr differenziert gesehen. Bereits die Unterscheidung von Rechts- und Linkspopulismus zeigt, dass unterschiedliche Ideologien in der Form des Populismus Verbreitung finden können (Hartleb 2004). Nicht notwendigerweise aber transportiert Populismus explizit Ideologien. Jagers und Walgrave unterscheiden hier zwischen einem „dicken“ Populismus, der ideologiebesetzt ist, und einem „dünnen“, der nur als Kommunikationsstil auftritt (Jagers und Walgrave 2007). Auch Priester spricht davon, dass Populismus weniger ein Substanz- als vielmehr ein Relationsbegriff ist (2007). Und Taggart, eine frühe Analytikerin des Populismus, hält ihn für „inhärent unvollständig“, für „ein leeres Herz“, ein „Chamäleon“ (2000). Demgegenüber wird jedoch kritisch eingewandt, dass nicht jedwede Theorie oder Ideologie oder politische Position populistisch sein kann (und darf), sondern dass er – auch wenn er nur als Kommunikationsstil verstanden wird – in sich antiliberale Züge trägt, ideologisches Substrat quasi in der Form mittransportiert (Lewandowsky 2011). Seine Grundmerkmale – Dichotomie von Volk und Elite, Ansprache von Emotion und Moral, Vereinfachung und Personalisierung, Polarisierung und Symbolisierung – sind mit zentralen und humanen Demokratiepostulaten inkompatibel. Andererseits: Kommunikationselemente des Populismus werden mittlerweile von nahezu allen demokratischen Parteien genutzt und sind im öffentlichen Diskurs durchaus legitim geworden.

Zur inhaltlichen Dimension des Populismus gehört auch die Frage nach der Wirklichkeit, der Realität, den Fakten. Natürlich ist ein Ansatz, der aus einer hochdifferenzierten Menge von Menschen ein homogenes Volk konstruiert, per se fragwürdig, was den Realitätsbezug und die Abbildung der Wirklichkeit betrifft. Allein dies erfordert immer wieder Umdeutungen von Realität oder – um es zeitnah zu formulieren – „alternative Fakten“. Gelingt es nicht, darüber die Vorstellung eines homogenen Volkes aufrecht zu erhalten, tritt das Verfahren der Polarisierung ein. Das Gegenüber, die Gegner, sind dann diejenigen, welche solche alternativen Fakten in Frage stellen, einen Riss geht durch das homogene Volk postulieren. Es entsteht – eine Folge der Polarisierung – eine Art ideologischer Bürgerkrieg, gut zu beobachten aktuell in den Auseinandersetzungen des US-amerikanischen Präsidentschaftswahlkampfs des Jahres 2020. Die Frage der Fakten spielt auch eine Rolle, weil in der Sicht populistischer Ansätze die Wissenschaft auf die „andere“ Seite gehört, abhängig von der politischen Machtelite und daher unglaubwürdig. Eine Differenzierung schlägt hier daher Jan-Werner Müller (2016) vor: Nicht der Inhalt oder die Form machen den Populismus aus, sondern die Begründung. Populistisch ist zu qualifizieren, was weder demokratisch legitimiert oder wissenschaftlich argumentiert ist.

Am häufigsten wird Populismus vom Nationalismus bedient – es ist „merely one of the ideologies that autocrats and populists utilize“ (Bieber 2018, S. 559), weil er die Homogenisierung ermöglicht, die stark auf dem Zugehörigkeitsgefühl basierende emotionale Komponente hat und in vielen quasi-wissenschaftlichen und pseudo-historischen Theorien die Begründung findet. Jenne and Csergö folgend nennt Bieber Ethnopopulismus „the global dynamics of nationalist mobilization“ und erklärt, wie sich Nationalismus und Populismus gegenseitig verstärken. Vor allem die gesellschaftlichen Gruppen, die sich als Verlierer der Globalisierungsprozesse sehen, neigen dazu, den Staat als „Verteidiger“ der nationalen Identität und der Werte zu sehen, die in der globalen, von den gierigen Kapitalisten dominierten Welt verloren gehen. Auch wirkt Nationalismus in vielen Bereichen „als Brandbeschleuniger, weil er Polarisierung fördert und damit Illiberalen in die Hände spielt“ (Lührmann und Hellmeier 2020).

Mit der demokratischen Legitimation erreichen wir den politischen Hintergrund des Populismus. Nicht immer, aber in den meisten Fällen setzen populistische Gedanken und Aktivitäten beim Willen des „Volkes“ an, der in der parlamentarischen und repräsentativen Demokratie nicht oder nicht mehr angemessen aufgehoben und abgebildet sei („democracy is on exile“). Zweifellos gibt es Kontexte und Konflikte, in denen dies der Fall ist, insbesondere in scheindemokratischen Gesellschaften. Die Parole „WIR sind das Volk“ beim Zusammenbruch des DDR-Regimes kann als populistisch bezeichnet, im Kontext aber kaum so bewertet werden; es überrascht jedoch nicht, dass diese Parole 25 Jahre später von AfD und Pegida benutzt wurde, um rechte Stimmungen zu generieren und aufzuheizen.

Im Kern jedoch richten sich, wenn ideologische Dimensionen zu erkennen sind, populistische Aktivitäten gegen Erscheinungs- und Funktionsweisen der repräsentativen Demokratie. „People the world over are rejecting the legitimacy of liberal democracy, hardening themselves against ‚enemies‘, retreating to the security of their tribe, and placing faith in populist leaders“ (Kennedy 2017).

Die Ursachen dafür liegen tief in der gesellschaftlichen Entwicklung insbesondere der letzten zwei Jahrzehnte. Vielfach wird diese Entwicklung unter dem Stichwort „Modernisierung“ zusammengefasst, eine Vokabel, die ähnlich euphemistisch ist wie der „Park“ für Industriegebiete. Unter „Modernisierung“ werden gewöhnlich die Prozesse und Ergebnisse der Globalisierung verstanden, die „Errungenschaften“ der Mikrotechnologie, die Veränderungen der Produktion hin zur Dienstleistungswirtschaft und die gewachsene räumliche, soziale und politische Mobilität der Bevölkerungen.

Die Bindung an traditionelle Parteien und Organisationen (Volksparteien, Kirchen, Gewerkschaften etc.) hat sich im selben Atemzug stark gelockert, Nachbarschaften und Familienstrukturen haben an Bedeutung verloren, neue Bindungen sind entstanden vor allem durch die sozialen Medien. In diesem Prozess der Modernisierung gibt es „Modernisierungsverlierer“, genau genommen besonders die „kleinen Leute“. „Populismus ist ein Modernisierungsphänomen“, schreibt Lewandowsky (2011, S. 222).

Populism is the voice of those who have already become or who fear becoming the victims of an economy which is less controlled and controllable by national governments than in the past (Pelinka 2008, S. 43).

Allerdings sind es keineswegs nur die Modernisierungsverlierer, die anfällig sind für populistische Vorgehensweisen. Es geht auch um Einstellungen und Werteparadigma. So zeigt sich etwa, dass die Befürwortung von Diktatur, Chauvinismus und Ausländerfeindlichkeit, Antisemitismus und Sozialdarwinismus sowie die Verharmlosung des Nationalsozialismus in allen gesellschaftlichen Schichten zu finden sind (Lewandowsky 2011, S. 225).

Mit seiner epistemologischen und moralischen Relativität, dem Hervorheben der menschlichen Subjektivität und der Mehrdeutigkeit der Interpretationen der Wahrheit sowie seiner Wissenschaftsskepsis hat er, der Postmodernismus, auch in der Erwachsenenbildung zu negativen Konsequenzen geführt. Freitag (2017) kritisiert etwa, dass ernsthaft behauptet wird, die Wissenschaft sei „nur Ausfluss der Herrschaftsideologie einer westlich dominierten Weltsicht“.

Auch wenn dies nicht die ganze Situation erfasst: die Wirksamkeit populistischer Strategien hängt konkret mit den Sorgen, Ängsten und Nöten von Menschen zusammen, die unsicher sind, ob die „herrschende“ politische Elite in den bestehenden Strukturen wirklich die Probleme lösen kann. Dabei spielt strukturell die Übermacht globaler Entwicklungen und Entscheidungen, die weitgehend undurchsichtig werden (der „Sumpf“, aus dem Verschwörungstheorien wachsen) ebenso eine Rolle wie die Abgehobenheit überstaatlicher Gebilde wie der Europäischen Union.

Wenn es denn hier einen Hebel gibt, den „Volkswillen“ in politisches Handeln umzusetzen, dann ist dies eine direkte Demokratie, sind das Volksentscheide und -abstimmungen. Darauf kaprizieren sich, wenn es um konkrete politische Handlungen geht, im Grunde die meisten populistischen Bewegungen oder Parteien. Allerdings: wenn es um umsetzbare Vorgehensweisen geht, hapert es in den populistischen Ansätzen, sie bleiben meist im Vagen. Nicht einmal Hinweise auf die Schweiz, wo in einigen Kantonen solche direkten Entscheidungen des Volkes erfolgen, sind in den Vorschlägen zu finden.

Geht es um Bildung, um organisierte und intentionale Bildungsarbeit, ist in Bezug auf den Populismus vor allem die „politische Bildung“ gefragt. Populismus ist wirksam in allen Belangen, die Gegenstand politischer Bildung sind. Für die politische Bildung an den Schulen stellt der Populismus eine Herausforderung dar. Dies liegt an den Curricula, aber auch an der inhaltlichen Varianz populistischer Ansätze und ihrer kontextabhängigen Erscheinungsform.

In der Erwachsenenbildung verstärkt sich das Problem. Das hängt auch zusammen mit dem Stand der politischen Erwachsenenbildung. Sie ist seit Jahrzehnten ein Stiefkind der Bildungspolitik. „Zweifellos hat der Verlust des möglichen Systemvergleichs zwischen Kapitalismus und Sozialismus bzw. Kommunismus die politische Bildung ihres wichtigsten Gegenstandes beraubt – das Gegenüberstellen unterschiedlicher Gesellschaftsmodelle war der zentrale Lebensnerv politischer Bildung“ (Nuissl 2007, S. 64). Politische Bildung gewann im Zuge der Migrations- und Flüchtlingswellen wieder an Bedeutung, aber meistens in Form von Integrationskursen für die Migranten, wobei die Bürgerinnen und Bürger der Aufnahmeländer selten involviert waren und sind. Nach den Terroranschlägen in Frankreich und Dänemark im Jahr 2015 reagierten europäische Minister mit einer Deklaration, die folgende Werte betont:

respect for human dignity, freedom (including freedom of expression), democracy, equality, the rule of law and respect for human rights. These values are common to the Member States in a European society in which pluralism, non-discrimination, tolerance, justice, solidarity and equality between women and men prevail (EU 2015, S. 1).

Die in Paris unterschriebene Deklaration wurde mit großer finanzieller Hilfe für die entsprechenden Projekte unterstützt (400 Mio. €, dazu 13 Mio. für die Dissemination und 14 Mio. für die *policy experimentation*; European Commission 2016). In der europäischen Bildungspolitik setzte sich immer mehr das Konzept *education for democratic citizenship *durch (oder *education for active citizenship*), womit neue Ansätze für die Bildungspolitik, neue Handlungsfelder, aber auch ein neuer Enthusiasmus angezielt waren und sind. Dennoch macht sich seit langem eine gewisse „Müdigkeit“ bemerkbar, die politische Bildung oder *civic education* als überflüssig oder sogar altmodisch einschätzt. Sie wird eher als Ausnahme betrieben und nicht als eine systematisch organisierte Bildungsintervention, die ernsthafte gesellschaftliche Probleme bearbeitet. „Politische Bildung, die sich der Wahrung und Entfaltung einer bindenden demokratischen Kultur verpflichtet weiß und die den Individuen die Fähigkeit und Bereitschaft zur umfassenden politischen Teilhabe ebenso vermitteln will wie die Kompetenz zur rationalen Bearbeitung, Beurteilung und Lösung gesellschaftlicher und politischer Schlüsselprobleme, scheint ihr Ziel mehr und mehr zu verfehlen“ (Wetterau 2000, S. 29).

Es geht hier um eine ungünstige Verflechtung – die Ursachen für die schon kontinuierliche Krise der politischen Erwachsenenbildung machen auch bei der Verbreitung des Populismus mit: starke Ökonomisierung der Bildung, das Fokussieren auf die berufliche Bildung und die Dominanz des gesellschaftlichen und wissenschaftlichen Diskurses, der auf Humankapitaltheorie beruht. Die *human capital theory* wurde unkritisch aus der Ökonomie übernommen und die dazu gehörenden Konzepte, Argumentationen und Zugänge auch.

Und das gilt nicht nur für Deutschland, sondern für die meisten westlichen Demokratien. Die Idee, die Demokratie durch die Bildungsarbeit zu stärken, scheint als *Fait accompli*, fast wie in Fukuyamas Rede vom „Ende der Geschichte“ (1992): Es entsteht der Eindruck, vor allem in den westlichen Ländern, als hätten sich Prinzipien des Liberalismus, der Demokratie und der Marktwirtschaft endgültig durchgesetzt.

Shin und Ging (2019, S. 13), die Erwachsenenbildungspolitik der USA kritisierend, behaupten, dass manche Maßnahmen „tactically legitimize(s) government’s neoliberal capitalist desire within a democratic society“, und Popović et al. (2020, S. 83) führen aus, dass „the neoliberal stream of the global development lead to somewhat reductive understanding of education and adult education, which is perceived mainly as the tool for economic development“.

Der aktuelle Diskurs wurde unter dem starken Einfluss der internationalen Akteure entwickelt, vor allem OECD und Weltbank, die nicht nur die Entwicklungszusammenarbeit auf die berufliche Bildung umorientierten, sondern eine starke globale Dominanz der Konzepte von *skills and competencies* initiiert haben. In den letzten zehn Jahren haben sich diese Konzepte auch in Europa fest etabliert (s. etwa „ET 2020“, „Europe 2020“ und „An agenda for new skills and jobs“; European Commission 2009, 2010a, b) bis zum neueren „Upskilling Pathways“; „European Skills agenda for competitiveness, social fairness and resilience“ und „Digital Education Action Plan“ (European Commission 2016, 2020a, b).

Die europäische Bildungspolitik ist durchdrungen von Begriffen wie „adaptability of the workforce“, „employability“ and „adaptability of citizens“, „human resource development“, „competitiveness“, „growth in the service of the knowledge-based economy“:

Education and training were also narrowed by the qualification-competence pairing, which also forbade the adoption of broader and more complex conceptions of AE, and the implementations of actions that encouraged the training of democratic, independent, thinking, and critical citizens (Lima und Guimaraes 2011, S. 109).

Ein späterer Versuch, *soft *und *interpersonal skills* in diese Konzepte zu integrieren, konnte den Bildungsfokus nicht wesentlich ändern, denn die Philosophie und das Menschenbild bei der so verstandenen beruflichen Bildung und der politischen Bildung bzw. *civic education* sind völlig unterschiedlich. Sie konnte nur eine Nische des Bildungspraxis, aber auch der Bildungsforschung bleiben. So finden sich heute selten Analysen und Programmvorschlägen über critical thinking, reflective thinking und meta-thinking.

Die globale Bildungs-Agenda wurden von denselben Faktoren bestimmt. Belegt ist dies in den Agenda 2030 (UN 2015), mit 17 Zielen der nachhaltigen Entwicklung (SDG), wo unter dem Ziel 4 (SDG4) politische Bildung bzw. *civic education* (im globalen Rahmen eher als *global citizenship education *konzipiert) nur unter dem Target 4.7 erwähnt wurde, zusammen mit vielen anderen:

By 2030, ensure that all learners acquire the knowledge and skills needed to promote sustainable development, including, among others, through education for sustainable development and sustainable lifestyles, human rights, gender equality, promotion of a culture of peace and non-violence, global citizenship and appreciation of cultural diversity and of culture’s contribution to sustainable development (UN 2015, Goal 4).

Die politische Gegenwart der globalen Welt ist kaum ein Thema, Populismus schon gar nicht. Sogar die „Incheon Declaration“ (UNESCO 2016), die das SDG 4 („inklusive, gleichberechtigte und hochwertige Bildung gewährleisten und Möglichkeiten lebenslangen Lernens für alle fördern“) operationalisiert, erwähnt nur in einem Paragraph eine Fähigkeit, die dem nahe kommt, die Lehrer besitzen sollten: „media literacy and source criticism skills“ (UNESCO 2016, S. 55). Konflikte und Krisen werden zwar betont, aber Populismus als ein globales Phänomen nicht.

Was die Politik nicht als wichtig erkennt, wird in der Bildungsforschung keine wesentliche Rolle spielen. Dies erfolgt nicht durch Verordnungen oder Bevormundung, sondern durch Anerkennung, Umsetzung und – Finanzierung. Mangel an Forschung ist die Widerspiegelung einer Realität, in der es wenig Interesse gibt, das Problem des Populismus zu lösen, sondern eher, es politisch zu instrumentalisieren. Es geht nicht nur um den Mangel an Unterstützung, die die Politik der Forschung geben soll, sondern mehr um die Diskurse und Paradigmen, welche alle gesellschaftlichen Bereiche bestimmen.

So wird in der Bildungsforschung die Dominanz der empirisch-quantitativen Methodologie evident. Der Druck, der Bildungspolitik praktisch brauchbare Ergebnisse zu liefern, damit eine *evidence-based policy* gemacht werden kann, lenkte die Aufmerksamkeit der Forschenden auf die Phänomene, die scheinbar sichtbar, greifbar und messbar sind. Die in der Erwachsenenbildung seit langem angewandten qualitativen und gemischt methodischen Ansätze (s. z. B. Grummell & Finnegan 2020) traten immer mehr in den Hintergrund. Die heute dominierenden Kriterien für die Evaluation der Forschungsleistungen^3^ vernachlässigen Forschungsergebnisse, die keine Zahlen als Grundlage haben.

Interessant ist die Meinung von Malesevic über die methodologischen Schwierigkeiten solcher Forschungen.

[He] raises the methodological question whether nationalism can begrasped through surveys. He argues convincingly that surveys are of limited value, asthe proxies used to measure the relevance of nationalism, such as pride in the nation, the exclusion of particular groups and other measures might effectively capture nationalism one context, but not in another (Bieber 2018, S. 561).

Dabei ermöglicht gerade die qualitative Forschungsmethode einen Zugang zum Verstehen des Populismus:

Basically, qualitative researchers are interested in understanding the meaning people have constructed; that is, how people make sense of their world and the experiences they have in the world […] The overall purposes of qualitative research are to achieve an understanding of how people make sense out of their lives, delineate the process (rather than the outcome or product) of meaning making, and describe how people interpret what they experience (Merriam und Tisdell 2016, S. 15).

Die Potenziale der qualitativen Forschung für den erwachsenenbildnerischen Zugang zum Populismus sind längst nicht ausgeschöpft.

Of course, surveys, census results and election outcomes can only offer a bird-eye view. The micro-processes of nationalism, from the everyday practices and the causal mechanisms of change do require other methods and more in-depth analysis. (Bieber 2018, S. 562)

Das Gleiche gilt für Populismus.

Nuissl erinnert daran, dass eine

Einigkeit besteht […] hinsichtlich der Auffassung, dass nicht mehr die großen theoretischen Entwürfe (Gesellschaftsmodelle und Gesellschaftstheorien) in der politischen Bildung relevant sind, sondern der Einzelne mit seinen Befindlichkeiten und Nöten (2007, S. 66).

Auch wenn sich das der Denkweise Fukuyamas nähert, verdienen die theoretischen Konzepte mehr Aufmerksamkeit, die sich den Menschen in ihrer komplexen Humanität widmen. Gerade in der politischen Bildung, die sich Themen wie Populismus, Radikalismus, soziale Bewegungen widmet, gibt es mittlerweile eine Reihe von Autoren mit Konzepten für eine neue Realität wie z. B. *public pedagogy* (Biesta et al. 2013, Biesta 2014; Giroux 2004, 2013; Ellsworth 2005). Große Aufmerksamkeit bekommen auch die Analysen von Noam Chomsky, Slavoljub Zizek und Naomi Klein, die systematisch und methodisch belegte Abhandlungen und Studien anbieten.

Diese Tendenzen führen dazu, dass Populismus, der als Phänomen nicht neu ist, aber dessen Kontext neue Untersuchungen benötigt, wenig Platz einnimmt in der traditionellen Bildungsforschung. Eine Ausnahme sind Statistiken und Umfragen über den Rechtspopulismus, die parteipolitischen Präferenzen und Einstellungen, Wahlen und schließlich Migranten. Aber eine Auseinandersetzung über mögliche Erwachsenenbildungsinterventionen – konzeptionell oder empirisch – ist immer noch ein Desiderat. Populismus ist schwer zu erfassen wegen der multidimensionalen Ursachen, der erforderlichen Interdisziplinarität (von Psychologie, über Soziologie, bis zur Politikwissenschaft), kaum erreichbarer Zielgruppen und komplexer Methodik ist der Populismus. Dies macht den Populismus für die Erwachsenenbildung in Praxis und Wissenschaft immer noch zu einer Herausforderung, aber auch zu einem immer wichtiger werdenden Thema.

Deshalb ist der wissenschaftliche Bedarf nach solchen Forschungen nur ein Aspekt des Problems. Wichtiger ist die Tatsache, dass die Vernachlässigung der Forschung zu bestimmten Themen zu einem Mangel an Verständnis und Konzepten führt, die Voraussetzung für zielgerichtetes Handeln sind.

Bei der politischen Bildung bzw. *civic education* ist Wissensvermittlung nur der Anfang, erst emotionale und kontextuale Komponenten bis hin zu Aktivitäten ergeben ein wirkliches Lernen.

Democratic societies need educated citizens who are steeped in more than the skills of argumentation. And it is precisely this democratic project that affirms the critical function of education and refuses to narrow its goals and aspirations to methodological considerations. This is what makes critical pedagogy different from training. And it is precisely the failure to connect learning to its democratic functions and goals that provides rationales for pedagogical approaches which strip the meaning of what it means to be educated from its critical and democratic possibilities (Giroux 2004, S. 502).

Wir, die Herausgeber der ZfW, hatten „Populismus“ mit dem Ziel ausgeschrieben, Licht in die Realität der politischen Bildung zum Populismus zu werfen, Untersuchungen zur Bildungsrealität des Phänomens zu sammeln und zu diskutieren. So lautete es im Call for Papers:

Political conditions call for deeper reflection of the role of adult education in the contemporary political and social framework. This refers to concepts of political education and education for democratic citizenship, to the assessment of their achievements and results until now, to the new understanding of media literacy with adult education, and – above all – to the possibility of strengthening critical and reflective thinking as the central effort of educationists facing the modern political changes.

In der Herleitung dieses Themas hatten wir darauf verwiesen, dass Populismus zwar schon immer existiert, heutzutage aber an Kraft und Wirksamkeit gewinnt. Dabei hatten wir als Charakteristika des Populismus Strategien der Polarisation, Personalisation, Moralisierung, verbunden mit Propaganda und Rhetorik, beschrieben. Als Gründe für das Erstarken des Populismus hatten wir angenommen, dass Menschen immer unzufriedener mit Politikerinnen und Politikern sowie der Politik an sich werden, dass sie diese als abgehoben und zu technokratisch empfinden. Dass sie eine Tendenz zu geringerer Gleichheit, zu Exklusion und Entfremdung sehen. Und dass sie drittens ein Gefühl der wachsenden Unsicherheit, Heimatlosigkeit und Erosion des sozialen Kapitals haben. Und dass hier den Pädagoginnen und Pädagogen, insbesondere denen in der politischen Erwachsenenbildung, eine besondere Aufgabe und Verantwortung zuwächst.

Wir haben viele Anfragen und Kommentare erhalten, letztlich aber wenige Forschungsbeiträge. Als Hauptursache sehen wir die obengenannte Ferne der Realität der politischen Bildung zu den aktuellen Paradigmen der (Erwachsenen‑)Bildungsforschung, aber auch die Marginalität, in die politische zugunsten der beruflichen Bildung gedrängt wurde und wird. Bereits dies also, denken wir, ist ein wichtiges Resultat der Themensetzung.

Die drei Beiträge des Schwerpunktthemas „Populismus und Erwachsenenbildung“ widmen sich unterschiedlichen Aspekten, was wiederum die Breite zeigt, in der Populismus wirksam ist.

Der erste Beitrag, von *Lukas Otterspeer und Christoph Haker*, thematisiert die Notwendigkeit, aber auch die Schwierigkeit einer bildungsmäßigen Auseinandersetzung mit dem Populismus, hier genauer: dem Rechtspopulismus. Die Autoren gehen davon aus, dass in einer einfachen Abgrenzung von Wissenschaft und Bildung auf der einen, Rechtspopulismus auf der anderen Seite kein geeigneter Ansatz der politischen Bildung liegt. Ganz im Gegenteil, das Schwarz-Weiß-Modell des Populismus wird damit letztlich verstärkt. Stattdessen schlagen sie den Ansatz des „boundary work“ vor, der wichtige Akzente in der epistemischen Diskussion setzt. Sie begründen ihren Zugang mit der Analyse des Falles einer rechtspopulistischen Organisation und Veranstaltung, und versuchen, daraus Grundlagen für eine kritische, aber auch handhabbare Position als Wissenschaftler und Lehrende gegenüber dem Rechtspopulismus zu entwickeln.

Der zweite Beitrag von *Thomas Wendt und Sebastian Markant* „konzipiert Populismus als gesellschaftsweit verbreitete Form der Kritik“ an einer Komplexität in Organisationen und grenzt ihn von anderen komplexitätsreduzierenden Deutungsmustern (wie Fundamentalismen und Verschwörungstheorien) ab. Dabei betrachten sie die weit verbreitete Kritik am Populismus ihrerseits kritisch:

An Zuschreibungen der Art, etwas sei populistisch, herrscht derzeit kein Mangel. Weniger klar ist, was Populismus genau meint. Vermutlich besteht hier ein Zusammenhang. Es scheint umso leichter, jemanden als Populisten zu bezeichnen, je weniger klar ist, was das bedeutet […] Populismus wird als ein Kommunikationsphänomen verstanden, das gesellschaftsweit auftritt, also auf grundlegende Zustände reagiert. Es handelt sich um eine Form der Kritik, die nicht allein im Feld der Politik und auch nicht erst in jüngerer Vergangenheit auftritt.

Mit diesem offenen Ansatz, dem Populismus eine kritische Rolle zuzuweisen, gelingt den Autoren ein nachdenklich machender Blick auf die Komplexität von Organisationen.

Der dritte Beitrag zum Schwerpunkt beschäftigt sich mit Gruppen, die nach Ansicht von Beobachtern und auch statistischen Analysen am anfälligsten für populistische Aktivitäten gelten: Menschen mit geringer Literalität. Der Autor, *Gregor Dutz*, untersucht auf der Basis der Daten aus der Studie LEO 2018 mit Hilfe logistischer Regressionsmodelle, ob diese Zielgruppe ihre eigenen politischen Kompetenzen gering einschätzt und sich daher vom politischen Feld fernhält. Im Ergebnis bestätigt sich diese Hypothese, mit der Konsequenz, dass in der Tat hier die Wahrscheinlichkeit einer Anfälligkeit für populistische Kommunikationsstile steigt.

Nicht ganz fern vom Thema Populismus liegt der Beitrag zum interkulturellen Training, verfasst von *Petia Genkowa und Amsy Whiting*. Sie gehen davon aus, dass mit der Globalisierung der Bedarf an interkultureller Kompetenz wächst. Folgerichtig bedarf es daher auch angemessener Maßnahmen, diese Kompetenz zu erwerben. Sie beschäftigen sich mit dem interkulturellen Training in der spezifischen Variante ohne begleitendes Coaching. Methodisch untersuchen sie Vergleichsgruppen, um die jeweilige Wirksamkeit festzustellen. Im Ergebnis stellen sie zwar verbesserte Herangehensweisen an Interkulturalität (etwa Verständnis, Kommunikation, Offenheit) bei den Teilnehmern an den Trainings fest, aber auch, dass das Training alleine noch keine interkulturelle Handlungskompetenz bewirkt, es sei daher eher als Ausgangspunkt für ein wirksameres interkulturelles Lernen zu sehen.

Die Autorinnen *Aiga von Hippel und Maria Stimm* erarbeiten in ihrem Beitrag ein Verfahren, wie Weiterbildungsinstitutionen weiter typisiert und ausdifferenziert werden können. Sie erweitern bestehende Typologien um die Kategorie der „beigeordneten“ Bildung und vermögen damit, ein breiteres Spektrum von Einrichtungen in systematischer Perspektive darzustellen. Sie verorten ihren Beitrag in einer Schnittstelle von Programm- und Organisationsforschung.

Der letzte Beitrag im Forum dieser Ausgabe schließlich, verfasst von *Andreas Seiverth*, hat eine Sonderstellung. Es handelt sich nicht um die Präsentation von Forschungsergebnissen, sondern um eher grundlegende methodologische Gedanken, die aus einer Rezension hervorgingen und in einem Essay zu Fragen der Geschichtsschreibung mündeten.

Ich möchte daher eine weiterführende Interpretationsperspektive vorschlagen, indem ich die Idee einer historisch-normativen Geschichtsschreibung (Erinnerungsarbeit) der Erwachsenenbildung zu skizzieren versuche. Ihr liegt die Intention und Überzeugung voraus, die sich jedoch erst durch die konkrete Rekonstruktionsarbeit als theoretische Voraussetzung begründen lässt, dass sich in den Institutionen und in der Praxis des Volksbildungswesens […] Ein normativer Gehalt verkörpert findet.

Seiner anregenden Gedanken wegen wurde dieser Essay von den Herausgebern der ZfW auch ohne Peer Review als eigenständiger Aufsatz angenommen. Möge auch dieser Beitrag Anlass für weitere Diskussionen nicht nur des rezensierten Buches sein – sondern auch für einen anhaltenden Diskurs über wissenschaftliche und gesellschaftliche Belange.

## Footnotes

1 Entstanden nach Donald Trumps Inauguration im Jahr 2017, von mehreren europäischen TV-Satiresendungen 2017–2019 produziert (http://everysecondcounts.eu/).

2 Rechtsextreme und Verschwörungsgläubige finden seit einigen Jahren auf „Telegram“ ein großes Publikum und ein Portal, das nicht, wie Facebook und Instagram, Falschmeldungen und menschenverachtende Kommentare sperrt. Dort dürfen sich bis zu 200.000 Mitglieder austauschen – bei WhatsApp sind es nur 256. Es treffen sich ausschließlich Anhänger bestimmter Meinungen, sie bleiben ohne Moderation unter sich. Auf diese Weise entstehen häufig „rechts-alternative Echokammern ohne jeglichen Widerspruch“, wie Blumenthaler erklärt. Die Folge ist ein „Filterblaseneffekt, der beeinflussbare Personen in eine alternative Wirklichkeit abrutschen lässt“. Diese verlernen, Aussagen zu hinterfragen, „da die Gegenreaktionen zu Falschinformationen und menschenfeindlichen Kommentaren nicht nur ausbleiben, sondern sich die Gruppen vermeintlich vertrauter Personen gegenseitig bestärken“ (Jugendschutz.net-Report: „Telegram: Zwischen Gewaltpropaganda und ‚Infokrieg‘“, 18.10.2020).

3 Dies ist ein Vorgang, der mittlerweile kommerzialisiert wird, wie von Thomson Reuters in „Hottest science of the year“.
